# Supplementary material for: Regulation of trophoblast fusion dysfunction in preeclampsia by the NCK1/EIF3D/PPARG axis
Source: Biol Res. 2026 Jun 10;59:46. doi: 10.1186/s40659-026-00704-y (PMC13479735; doi:10.1186/s40659-026-00704-y)
Supplement: Supplementary file 1 [file 40659_2026_704_MOESM1_ESM.docx]

**Regulation of Trophoblast Fusion Dysfunction in Preeclampsia by the NCK1/EIF3D/PPARG Axis**

Ying Shen^1#^; Xiaolin Gao^2#^; Jiashi Wang^3*^; Yingying Hao^1*^

1. Department of Obstetrics and Gynecology, Shengjing Hospital of China Medical University, Shenyang, 110004, People's Republic of China

2. Department of Urology, The Air Force Hospital of Northern Theater PLA, Shenyang, 110042, People's Republic of China

3. Department of Orthopedics, Shengjing Hospital of China Medical University, Shenyang, 110004, People's Republic of China

^#^The authors contributed equally

***Corresponding author**

Dr. Yingying Hao, E-mail: [haoyy_doc@163.com](mailto:haoyy_doc@163.com)

Department of Obstetrics and Gynecology, Shengjing Hospital of China Medical University, No. 36 Sanhao Street, Heping District, Shenyang, 110004, People's Republic of China

Dr. Jiashi Wang, E-mail: [wangjiashi2018@163.com](mailto:wangjiashi2018@163.com)

Department of Orthopedics, Shengjing Hospital of China Medical University, No. 36 Sanhao Street, Heping District, Shenyang, 110004, People's Republic of China

**Abstract**

**Background:** Abnormal syncytialization of cytotrophoblast (CTB) cells is known to be associated with preeclampsia (PE), however, the underlying molecular mechanisms remain elusive.

**Results:** In this study, proteomic analysis of placental tissues was performed to identify proteins involved in PE pathogenesis. NCK Adaptor Protein 1 (NCK1) was found to be downregulated in placental tissues from patients with early-onset PE. *In vitro* functional assays revealed that NCK1 was markedly increased during spontaneous syncytialization of CTB cells. NCK1 overexpression alleviated hypoxia-induced defects in CTB cell fusion. Mechanistically, NCK1 upregulated both the expression and stability of Peroxisome Proliferator-Activated Receptor Gamma (PPARG), accompanied by increased m6A modification of its mRNA. Overexpression of PPARG significantly rescued hypoxia-induced syncytialization impairment, whereas PPARG deficiency abolished the promotive effects of NCK1 on CTB syncytialization. Additionally, phosphorylation of Eukaryotic Translation Initiation Factor 3 Subunit (EIF3D) was significantly increased during spontaneous syncytialization, and NCK1 overexpression partially reversed hypoxia-induced suppression of EIF3D phosphorylation. EIF3D knockdown significantly reduced m6A levels on PPARG mRNA, likely due to impaired NCK1-mediated suppression of Alpha-Ketoglutarate-Dependent Homolog 5 (ALKBH5), an m6A demethylase. Furthermore, NCK1 enhanced EIF3D phosphorylation by inhibiting its O-GlcNAcylation.

**Conclusions:** Our findings demonstrate that NCK1 stabilizes PPARG by modulating the crosstalk between O-GlcNAcylation and phosphorylation of EIF3D, thereby restoring hypoxia-impaired syncytialization in CTB cells. This study identifies the NCK1/EIF3D/PPARG axis as a molecular pathway potentially relevant to trophoblast fusion dysfunction in PE.

**Keywords**: EIF3D, NCK1, PPARG, m6A, O-GlcNAcylation, preeclampsia

**Background**

Preeclampsia (PE) is a placental disorder characterized clinically by new-onset hypertension and proteinuria [1]. Clinically, PE is classified into two well-established subtypes: early-onset PE (gestational age < 34 weeks) and late-onset PE (≥ 34 weeks). Early-onset PE is primarily associated with defective placental development, whereas late-onset PE is largely attributed to interactions between normal placental senescence and a maternal genetic predisposition to cardiovascular or metabolic disorders [2].

During placentation, mononucleated cytotrophoblast (CTB) cells either differentiate into invasive extravillous trophoblasts or fuse to form multinucleated syncytiotrophoblasts (STB), which constitute the epithelial covering of placental villi in direct contact with maternal blood [1, 3]. Under pathological stress, such as placental ischemia or chronic hypoxia, STB cells undergo inflammatory activation, leading to the release of pro-inflammatory cytokines, anti-angiogenic factors, and cell-free fetal DNA into the maternal circulation [3, 4]. These factors contribute to systemic endothelial dysfunction and a maternal inflammatory response, which manifest as the clinical syndrome of PE. In early-onset PE, defective remodeling of the uterine spiral arteries leads to uteroplacental malperfusion [5]. This hemodynamic insufficiency causes persistent STB injury, resulting in STB dysfunction. Thus, a systematic investigation of the regulatory mechanisms underlying STB formation is of significant clinical value.

NCK Adaptor Protein 1 (NCK1), a signaling protein containing Src homology 2 and 3 (SH2 and SH3) domains [6], plays a crucial role in mediating signal transduction, including growth factor responses, immune cell activation [7, 8], and cancer metastasis [9]. Recent studies have shown that NCK1 is significantly reduced in both peripheral blood mononuclear cells (PBMCs) and decidual tissues of patients with recurrent miscarriage [10]. Moreover, knockdown of NCK1 inhibited trophoblast cell proliferation and migration, suggesting a potential role for NCK1 in pregnancy maintenance.

Eukaryotic Translation Initiation Factor 3 Subunit (EIF3D) is a non-canonical mRNA cap-binding protein that mediates an alternative mechanism of mRNA translation initiation [11]. EIF3D has been reported to be upregulated in placental tissues from patients with PE [12], and its overexpression promotes trophoblast proliferation and invasion, thereby exacerbating PE [12]. Mukhopadhyay et al. [13] demonstrated that dephosphorylation of EIF3D significantly upregulates Alpha-Ketoglutarate-Dependent Homolog 5 (ALKBH5), an m6A demethylase. Inhibition of ALKBH5 enhances the mRNA stability of Peroxisome Proliferator-Activated Receptor Gamma (PPARG), which in turn suppresses oxidative stress in trophoblasts and alleviates PE progression [14]. Notably, PPARG is essential for placental development [15], and its knockdown impairs trophoblast fusion [15]. However, the molecular mechanisms by which PPARG regulates CTB differentiation remain poorly understood.

In this study, we report for the first time that NCK1 expression is reduced in placental tissues from patients with early-onset PE. Furthermore, we demonstrate that NCK1 regulates the phosphorylation of EIF3D via O-GlcNAcylation, thereby modulating PPARG expression. Collectively, our findings suggest that dysregulation of the NCK1/EIF3D/PPARG signaling axis may contribute to trophoblast fusion defects in PE pregnancies.

**Methods**

**Human placental tissue**

A total of 10 term placentas were collected from healthy pregnant women, and 10 placentas were collected from patients with early-onset PE (gestational age 23–33 weeks). Among the normal placentas, one was used for the isolation of primary CTB cells, and the remaining nine were snap-frozen for label-free proteomic analysis, immunoblotting, and immunohistochemistry experiments. Four PE placentas were subjected to label-free proteomic analysis, and the remaining six were used for immunoblotting to assess NCK1 expression in placental tissues. All placental samples were collected after informed consent was obtained from the participants at Shengjing Hospital of China Medical University.

**Label-free proteomic analysis of placental tissues**

Placental tissues were ground in liquid nitrogen and subsequently homogenized in 8 M urea lysis buffer supplemented with protease inhibitors at a 50:1 ratio, followed by thorough vortexing. After homogenization, the lysates were clarified by centrifugation at 14,000 × g for 20 min, and the supernatants containing extracted proteins were stored at -80°C until further analysis. Protein concentrations were determined using the Bradford assay. According to the manufacturer’s protocol, protein samples were purified using a Proteomic Digestion Kit (QLBIO MagicOmics-MMB8X, Beijing Qinglian Biotech Co., Ltd., Beijing, China) to remove residual salts and small-molecule contaminants, and subsequently stored at -80°C until loading. Nanoflow LC-MS/MS analysis of the tryptic peptides was performed on a quadrupole Orbitrap mass spectrometer (Q Exactive HF-X, Thermo Fisher Scientific, Bremen, Germany). Briefly, a total of 500 ng of peptides was loaded onto a 25 cm column with a 150 μm inner diameter (Beijing Qinglian Biotech Co., Ltd.). Peptides were separated using a gradient of 8–12% solvent B (80% acetonitrile, 0.1% formic acid in water) over 5 min, 12%–30% B over 33 min, increased to 40% B over 7 min, and followed by a 15 min wash at 95% B, with a flow rate of 600 nL/min. The total run time was 1 h. The mass spectrometer was operated in a “top-40” data-dependent mode. MS spectra were acquired in the Orbitrap mass analyzer at a resolution of 120,000 over a 350-1500 m/z range. MS/MS spectra were collected in the Orbitrap at a resolution of 15,000 following higher-energy collisional dissociation (HCD) with a normalized collision energy (NCE) of 27.

**Isolation of primary CTB cells**

Primary CTB cells were isolated from normal human term placentas according to the protocol by Gargi Jaju Bhattad et al. [16], with minor modifications. Briefly, placental tissues were thoroughly washed with PBS, minced, and digested in digestion buffer [Hank’s solution supplemented with 1M HEPES, 7.5% sodium bicarbonate, 2.5% trypsin (Solarbio Science & Technology, Co., Ltd., Beijing, China) and 150 kU DNase I (Biosharp, Anhui, China)] at 37°C for 30 min. The resulting cell suspension was collected and resuspended in fetal bovine serum (FBS) to terminate enzymatic activity. After filtering through a 100 μm strainer, the cell pellet was resuspended in DMEM/F12 medium (Biosharp) containing 10% FBS and layered onto a pre-prepared Percoll gradient (5-70%, Biosharp), followed by centrifugation at 1200 × g for 20 min. Cells located at the interface between the 45% and 35% Percoll layers were collected, resuspended in DMEM/F12 medium, and centrifuged at 350 × g for 10 min. To remove non-CTB cells, 1 × 10^7^ cells were resuspended in 100 μl separation buffer and incubated with 10 μl of phycoerythrin-conjugated anti-human leukocyte antigen-ABC antibody (Miltenyi Biotec, Germany) for 10 min at 4°C. After washing, the cells were resuspended in 80 μl buffer and incubated with 20 μl anti-phycoerythrin-coated microbeads (Miltenyi Biotec) at 4°C for 15 min. Following another wash step, the cells were resuspended in 500 μl buffer, and target cells were isolated using magnetic-activated cell sorting. Finally, the isolated cells were cultured in DMEM/F12 medium (Biosharp) supplemented with 10% FBS at 37°C in a 5% CO₂ incubator.

**Plasmid and lentiviral vector construction**

Full-length NCK1 or PPARG cDNA were cloned into the pLVX-IRES-puro lentiviral vector (Cat No. BR025, Hunan Fenghui Biotechnology Co., Ltd., Hunan, China) using XhoI/NotI restriction sites. Target-specific shRNA sequences for PPARG (5’-CAGCATTTCTACTCCACATTA-3’, shPPARG) and EIF3D (5’-GACGACATGGATAAGAATGAA-3’, shEIF3D) were designed and cloned into the pLVX-shRNA1 lentiviral vector (Cat No. BR004, Hunan Fenghui Biotechnology Co., Ltd.) via BamHI/EcoRI restriction sites. A scrambled shRNA (5’-TTCTCCGAACGTGTCACGT-3’) was used as a negative control. To generate the EIF3D expression plasmid, full-length EIF3D cDNA was cloned into the pcDNA3.1-3×flag vector (YouBio, Hunan, China) using BamHI/XhoI restriction sites. Similarly, full-length OGT cDNA was inserted into the pcDNA3.1-Myc vector (GeneralBiol, Anhui, China) using NheI/EcoRV restriction sites, and full-length NCK1 cDNA was cloned into the pcDNA3.1-HA vector (GeneralBiol) using BamHI/XhoI restriction sites. The efficiency of overexpression and knockdown was verified by quantitative real-time PCR (qPCR) and immunoblotting assays.

**Immunohistochemistry**

Fixed placental tissues were thoroughly washed under running water, dehydrated through a graded ethanol series (70%–100%), cleared in xylene for 30 min, and embedded in paraffin wax. Tissue sections (5 μm thick) were prepared, deparaffinized in xylene, and rehydrated through a graded ethanol series (95%, 85% and 75%) followed by distilled water. Antigen retrieval was performed by heat treatment using a microwave oven, and endogenous peroxidase activity was blocked with 3% H_2_O_2_. After rinsing with PBS, the sections were blocked with 1% BSA at room temperature for 15 min. Subsequently, sections were incubated overnight at 4°C with primary antibodies against cytokeratin 7 (CK7, Cat No. A4357, 1:100, ABclonal Biotechnology Co., Ltd., Shanghai, China) and NCK1 (Cat No. 15247-1-AP, 1:100, Proteintech Group, Inc., Wuhan, China). After rinsing five times with PBS, the sections were incubated with HRP-conjugated goat anti-rabbit IgG secondary antibody (Cat No. D110058, 1:200, Sangon Biotech, Shanghai, China) for 1 h at room temperature. Then, 3,3’-diaminobenzidine (DAB) solution was added to produce a brown precipitate at the site of antibody binding, followed by hematoxylin counterstaining. Finally, the sections were dehydrated through a graded ethanol series (75%, 85%, and 95%), cleared in xylene for 10 min, mounted, and imaged using a fluorescence microscope (Olympus, Japan).

**Immunofluorescence assay of E-cadherin protein**

CTB cells were fixed in 4% paraformaldehyde for 15 min. After washing three times with PBS, the cells were permeabilized with 0.1% Triton X-100 (Beyotime Biotech Co., Ltd., Shanghai, China) for 30 min, washed thoroughly with PBS, and then blocked with 1% BSA for 15 min. Subsequently, the cells were incubated with a primary antibody against E-cadherin (Cat No. A20798, 1:100, ABclonal Biotechnology Co., Ltd.) overnight at 4°C. After rinsing five times with PBS, the cells were incubated with Alexa Fluor^®^ 555-conjugated anti-rabbit IgG secondary antibody (Cat No. 4413S, 1:200, Cell Signaling Technology, Inc., USA) for 1 hour at room temperature. The nuclei were then counterstained with DAPI (Aladdin, Shanghai). Finally, images were captured using a fluorescence microscope (Olympus, Japan).

**qPCR**

Total RNA from primary CTB cells and lentivirus-infected CTB cells was isolated using TRIpure reagent (BioTeke Corporation, Beijing, China) according to the manufacturer’s instructions. RNA quality and concentration were assessed using a NanoDrop One UV-Vis Spectrophotometer (Thermo Fisher Scientific, MA, USA). Subsequently, cDNA was reverse-transcribed from RNA using the All-in-One First-Strand SuperMix Kit (Guangzhou Magen Biotechnology Co., Ltd., Guangzhou, China). qPCR was performed using SYBR Green I (0.5 μl, Solarbio Science & Technology, Co., Ltd.) in a 20 μl reaction system containing 1 μl cDNA, 0.5 μM of each primer, and 10 μl of 2 × Fast Taq plus PCR Master Mix (Biosharp). β-actin was used as the internal control. Relative gene expression levels were calculated using the 2^−ΔΔCt^ method. The following primers were used: ERVW1 Forward, 5’-CCCTTACCAAGAGTTTC-3’; ERVW1 Reverse, 5’-AAGAGTGGCAGAGTGAT-3’. ERVFRD-1 Forward, 5’-GGCAGCACAGGGAGGAAT-3’; ERVFRD-1 Reverse, 5’-CGTAAACTGGAGGCTTGAT-3’. CGB Forward, 5’-CCTGGCCTTGTCTACCTCTT-3’; CGB Reverse, 5’-GGCTTTATACCTCGGGGTTG-3’. PPARG Forward, 5’-CAGGAGCAGAGCAAAGA-3’; PPARG Reverse, 5’-CTCGGATATGAGAACCC-3’. β-actin Forward, 5’-GCACAGAGCCTCGCCTT-3’; β-actin Reverse, 5’-CCTTGCACATGCCGGAG-3’.

**β-human chorionic gonadotropin (β-hCG) ELISA assay**

Cell supernatants were collected according to the experimental design. Secreted β-hCG was quantified using a Human β-HCG ELISA Kit (Wuhan Fine Biotech Co., Ltd., Wuhan, China), following the manufacturer’s instructions.

**Protein purification, co-immunoprecipitation (Co-IP), and immunoblotting**

Placental tissues and the indicated CTB cells were lysed using cell lysis buffer (Beyotime Biotech Co., Ltd.) supplemented with the protease inhibitor phenylmethanesulfonyl fluoride (PMSF, Beyotime Biotech Co., Ltd.). Protein concentrations were determined using a BCA Protein Assay Kit (Beyotime Biotech Co., Ltd.).

For immunoprecipitation, cell lysates were incubated with the indicated primary antibodies using a Pierce™ Co-IP Kit (Thermo Fisher Scientific) at 4°C overnight, following the manufacturer’s protocol. The immunoprecipitated proteins were subsequently analyzed by immunoblotting.

For immunoblotting, equal amounts of protein (20–40 μg) in 20 μl lysate were separated by 10% SDS-PAGE at 80 V for 2.5 h and then transferred onto polyvinylidene difluoride membranes. The membranes were blocked with QuickBlock™ Blocking Buffer (Beyotime Biotech Co., Ltd.) for 1 h at room temperature and incubated overnight at 4°C with the following primary antibodies: NCK1 antibody (Cat No. 15247-1-AP, Proteintech Group, Inc.), β-hCG antibody (Cat No. A24128, ABclonal Biotechnology Co., Ltd.), PPARG antibody (Cat No. 16643-1-AP, Proteintech Group, Inc.), EIF3D antibody (Cat No. 10219-1-AP, Proteintech Group, Inc.), ALKBH5 antibody (Cat No. 16837-1-AP, Proteintech Group, Inc.), pSer/Thr (Cat No. PP2551, ECM Biosciences, KY, USA), O-GlcNAc (Cat No. MA1-038, Thermo Scientific), Flag (Cat No. 66008-4-Ig, Proteintech Group, Inc.), Myc (Cat No. 16286-1-AP, Proteintech Group, Inc.), HA (Cat No. 51064-2-AP, Proteintech Group, Inc.) or β-actin (Cat No. sc-47778, Santa Cruz Biotechnology, Inc., CA, USA). After washing three times with Western washing buffer (10×), the membranes were incubated with the appropriate HRP-conjugated goat anti-rabbit IgG (Cat No. A0208, Beyotime Biotech Co., Ltd.) or Goat Anti-Mouse IgG (Cat No. A0216, Beyotime Biotech Co., Ltd.) secondary antibodies for 1 hour at room temperature. Protein bands were visualized using the BeyoECL Plus Kit (Beyotime Biotech Co., Ltd.), and band densities were quantified using Gel-Pro Analyzer software.

**Analysis of O-GlcNAcylated EIF3D**

O-GlcNAcylation of EIF3D was analyzed using an enzymatic labeling method as previously described [17]. Briefly, cell lysates were labeled according to the manufacturer’s instructions for the Click-iT O-GlcNAc Enzymatic Labeling System (Thermo Fisher Scientific). The labeled proteins were then conjugated with an alkyne-biotin compound using the Click-iT Protein Detection Kit (Thermo Fisher Scientific). Subsequently, biotinylated proteins were precipitated using streptavidin resin. The levels of EIF3D in both the eluted fraction and input samples were analyzed by immunoblotting, and band intensities were quantified using Gel-Pro Analyzer software.

**Statistical analysis**

All data are presented as the mean ± SD from at least three independent biological replicates. The results were analyzed using GraphPad Prism software (GraphPad Software, Inc., California, USA). Student’s t-test was used for comparisons between two groups, and ordinary one-way ANOVA with Tukey’s multiple comparisons test was performed for comparisons among multiple groups. *P* < 0.05 was considered statistically significant.

**Results**

**Reduced NCK1 expression in placental tissues from early-onset PE patients**

In this study, placental tissues from early-onset PE patients (n = 4) and healthy controls (n = 4) were subjected to LC-MS/MS-based label-free proteomic analysis. Principal component analysis (PCA) revealed a clear separation between PE and control samples (**Fig. 1A**). A total of 25 differentially expressed proteins (DEPs), including 15 up-regulated and 10 down-regulated proteins (|log2 FC| > 1, *P* < 0.01), were identified in PE placentas (**Fig. 1B**). Gene Ontology biological process (GO-BP) enrichment analysis of the top 15 terms highlighted three key proteins: NCK1, Three Prime Repair Exonuclease 1 (TREX1), and Cathepsin A (CTSA). Notably, NCK1 was significantly enriched in endoplasmic reticulum (ER) stress-related processes (**Fig. 1C**). Given that ER stress has become a central component of the placental pathology underlying PE [18, 19], these findings suggest a potential role for NCK1 in PE pathogenesis. KEGG pathway analysis further indicated that NCK1 is involved in the ErbB signaling pathway (**Fig. 1C**), which has been implicated in trophoblast dysfunction in PE [20]. To further identify candidate genes associated with trophoblast fusion, we queried the GeneCards database (https://www.genecards.org/) using the term “trophoblast fusion”. Intersection analysis between the proteomic dataset and GeneCards results identified 13 overlapping candidates (**Fig. 1D**). Among these, four proteins were down-regulated, and notably, only NCK1 has not been previously reported in the context of PE. Finally, immunoblotting confirmed that NCK1 expression was significantly decreased in PE placental tissues compared with controls (**Fig. 1E**). Together, these findings prompted us to further investigate the role of NCK1 in trophoblast syncytialization and its underlying molecular mechanisms.

**NCK1 is upregulated during CTB cell fusion**

Next, immunohistochemistry was performed to evaluate the localization of NCK1 in human placenta, and the results showed that NCK1 was expressed in both CTB cells (CK7-positive) and STB cells (syncytial knots with obvious nuclear aggregation, **Fig. 2A**). To further elucidate the role of NCK1 in the spontaneous syncytialization of primary CTB cells, CTB cells were isolated from normal human term placentas and cultured for 5 days. As shown in **Fig. 2B**, CTB cells at day 0 predominantly existed as mononuclear cells (arrow). By day 5, CTB cells spontaneously fused to form multinucleated STB cells (dashed areas), as evidenced by the presence of multiple nuclei (DAPI, blue) enclosed within a continuous cell membrane boundary marked by E-cadherin (red). In the corresponding bright-field images, CTB cells at day 0 displayed a typical small, round morphology with clear cell boundaries, whereas at day 5, fused cells exhibited flattened and irregular shapes with indistinct cell borders (dashed areas), consistent with the formation of syncytial structures. As a marker for trophoblast syncytialization, the increased secretion levels of β-hCG (**Fig. 2C**) and its elevated mRNA expression (encoded by CGB, **Fig. 2D**) also confirmed the spontaneous syncytialization of CTB cells. Meanwhile, the elevated mRNA levels of syncytin-1 (encoded by ERVW1, **Fig. 2E**) and syncytin-2 (encoded by ERVFRD-1, **Fig. 2F**) further corroborated the successful establishment of this model. Notably, we observed that NCK1 was significantly upregulated during the syncytialization process (**Fig. 2G**), implicating its involvement in human placental syncytialization.

**Overexpression of NCK1 alleviates hypoxia-induced CTB cell fusion defect**

Given the established association between chronic hypoxia and PE pathogenesis, we exposed primary CTB cells to normoxia (21% O_2_, 5% CO_2_) or hypoxia (2.5% O_2_, 5% CO_2_) conditions for 5 days, according to a previous study [5]. As shown in **Fig. 3A**, the dashed areas in both the fluorescence and corresponding bright-field images indicated that hypoxic exposure significantly disrupted CTB syncytialization. Consistently, β-hCG protein (**Fig. 3B**) and the mRNA levels of β-hCG (encoded by CGB, **Fig. 3C**), syncytin-1 (encoded by ERVW1, **Fig. 3D**) and syncytin-2 (encoded by ERVFRD-1, **Fig. 3E**) were downregulated. In subsequent experiments, β-hCG expression was assessed by qPCR and Western blotting only, as the initial measurements in **Fig. 2C** and **2D** already confirmed that changes in secretion levels closely corresponded with mRNA expression, and were therefore considered sufficient to reflect its expression dynamics under hypoxia. Interestingly, NCK1 expression was also decreased after 5 days of culture under hypoxia (**Fig. 3F**). To investigate the role of NCK1 in CTB syncytialization under hypoxia, we infected primary CTB cells with lentivirus expressing NCK1 (oeNCK1, **Fig. 3G**). As shown in IF assays (**Fig. 3H**), overexpression of NCK1 significantly alleviated hypoxia-induced syncytialization impairment in CTB cells, as evidenced by increased formation of multinucleated STB cells (dashed area), elevated β-hCG protein expression (**Fig. 3I**), and upregulated mRNA levels of β-hCG (encoded by CGB, **Fig. 3J**), syncytin-1 (encoded by ERVW1, **Fig. 3K**) and syncytin-2 (encoded by ERVFRD-1, **Fig. 3L**).

**Overexpression of NCK1 upregulates PPARG expression and stability**

Although PPARG is known to play an essential role in placental development [15], its molecular mechanisms regulating CTB cell differentiation are still unclear. As expected, increase PPARG expression was observed in the spontaneous syncytialization process of primary CTB cells (**Fig. 4A**). Compared with normoxic culture, PPARG expression decreased after 72 hours of hypoxia but was partially rescued by NCK1 upregulation (**Fig. 4B** and **4C**). Furthermore, **Fig. 4D** showed that NCK1 stabilized PPARG mRNA, as evidenced by reduced transcript decay following actinomycin D treatment. The MeRIP results also showed that overexpression of NCK1 significantly increased the enrichment of PPARG mRNA in the anti-m6A immunoprecipitates (CTB-oeNCK1 *vs*. CTB-vec group, **Fig. 4E**), suggesting that NCK1 might regulate PPARG mRNA expression in an m6A-dependent manner.

**NCK1 regulates CTB cell fusion in a PPARG-dependent manner**

Next, we found that PPARG expression was significantly reduced in CTB cells cultured under hypoxic conditions (**Fig. 5A**). Thus, primary CTB cells with enhanced PPARG expression (oe-PPARG, **Fig. 5A**) were constructed to investigate its role in CTB syncytialization. As shown in IF assays (**Fig. 5B**), overexpression of PPARG significantly alleviated hypoxia-induced syncytialization impairment in CTB cells, as evidenced by increased multinucleated STB cells (dashed area), upregulated mRNA level of syncytin-2 (encoded by ERVFRD-1, **Fig. 5C**), and elevated β-hCG protein expression (**Fig. 5D**). Furthermore, to investigate whether PPARG mediates the effect of NCK1 on CTB syncytialization, a PPARG-knockdown lentiviral vector (shPPARG) was constructed. CTB cells were then co-infected with the shPPARG vector and a lentivirus overexpressing NCK1 (oeNCK1). After 48 hours, the cells were cultured under hypoxic conditions for an additional 72 hours. qPCR analysis showed that NCK1 overexpression significantly upregulated the expression of PPARG (**Fig. 5E**) and syncytin-2 (encoded by ERVFRD-1, **Fig. 5F**), whereas PPARG knockdown partially attenuated this effect. Consistent changes in β-hCG protein levels were confirmed by immunoblotting analysis (**Fig. 5G**). In line with these findings, IF staining and the corresponding bright-field images demonstrated that PPARG knockdown markedly suppressed the formation of multinucleated STB cells (dashed area) induced by NCK1 overexpression (**Fig. 5H**). Collectively, these results indicate that NCK1 regulates CTB cell fusion in a PPARG-dependent manner.

**NCK1 regulates PPARG expression by modulating EIF3D phosphorylation**

As shown in **Fig. 6A**, compared with Day 0, the Ser/Thr phosphorylation level of EIF3D was significantly increased at Day 5. Meanwhile, the enrichment efficiency of EIF3D in the immunoprecipitates was comparable between groups. In addition, no significant difference in total EIF3D expression was observed in whole-cell lysates (Input) between Day 0 and Day 5, indicating that EIF3D phosphorylation is enhanced during spontaneous syncytialization of primary CTB cells. Given the established role of NCK1 in modulating phosphorylation of downstream proteins (e.g., PERK) [21], we further investigated whether NCK1 regulates EIF3D phosphorylation during CTB syncytialization. As shown in **Fig. 6B**, EIF3D phosphorylation was significantly reduced in CTB cells cultured under hypoxic conditions, while overexpression of NCK1 partly rescued the phosphorylation levels of EIF3D. Previous studies have shown that dephosphorylation of EIF3D upregulate the expression of ALKBH5, an m6A demethylase [13]. Given that ALKBH5 knockdown enhances the mRNA stability of PPARG [14], we hypothesized that NCK1 might control PPARG stability by altering EIF3D phosphorylation. To verify this, EIF3D knockdown CTB cells were first generated using shEIF3D (**Fig. 6C**). Then, the indicated cells were treated with actinomycin D (5 μg/ml) after culture under hypoxic conditions. The results showed that NCK1 overexpression enhanced PPARG mRNA stability under hypoxic conditions, while knockdown of EIF3D suppressed this effect (**Fig. 6D**). In addition, the MeRIP results showed that knockdown of EIF3D significantly inhibited the enrichment of PPARG mRNA in the anti-m6A group [CTB (oeNCK1 + shNC) *vs*. CTB (oeNCK1 + shEIF3D) group, **Fig. 6E**], which might be attributed to EIF3D knockdown impairing NCK1-mediated suppression of ALKBH5 (**Fig. 6F**).

**NCK1 regulates the phosphorylation of EIF3D via O-GlcNAcylation**

Given the known crosstalk between O-GlcNAcylation and phosphorylation, and the predicted O-GlcNAcylation sites on EIF3D (NetOGlyc database, <https://services.healthtech.dtu.dk/services/NetOGlyc-4.0/>) [22], we further investigated the O-GlcNAcylation of EIF3D in CTB cells. Firstly, we found a significant decrease in O-GlcNAcylation of EIF3D during spontaneous syncytialization of primary CTB cells (**Fig. 7A**). To determine whether NCK1 affects the O-GlcNAcylation of EIF3D, we performed a chemoenzymatic labeling experiment as previously reported [17] (**Fig. 7B**). Immunoblotting analysis showed detectable signals in the eluted fraction of experimental group but not in the control group (CTB), which provided evidence that EIF3D was indeed O-GlcNAcylated. Overexpression of NCK1 decreased the immunoblotting signals of O-GlcNAcylated EIF3D in eluted fraction (**Fig. 7B**). Further Co-IP assays also showed that hypoxia led to increased O-GlcNAcylation of EIF3D, whereas decreased O-GlcNAcylation of EIF3D was observed upon NCK1 overexpression (**Fig. 7C**). Similar results were also observed in HEK293T cells (exogenous validation, **Fig. 7D**), indicating that NCK1 inhibited the O-GlcNAcylation of EIF3D in cells. These interesting results then led us to ask whether NCK1-mediated phosphorylation of EIF3D correlates with its O-GlcNAcylation. As shown in **Fig. 7E**, we transfected HEK293T cells with the indicated plasmids, and immunoprecipitated Flag-tagged EIF3D from the cell lysates. The use of a general pSer/Thr antibody revealed a robust, O-GlcNAc transferase (OGT)-mediated decrease in levels of EIF3D phosphorylation (**Fig. 7E**). However, the reduced EIF3D phosphorylation induced by OGT was reversed upon NCK1 overexpression.

**Discussion**

Currently, defective placental development induced by impaired spiral artery remodeling is widely recognized as a major cause of early-onset PE [23]. In this study, we found that NCK1 was downregulated in placental tissues from patients with early-onset PE. Overexpression of NCK1 alleviated hypoxia-induced defects in CTB cell fusion in a PPARG-dependent manner. Furthermore, we demonstrated that EIF3D is required for NCK1-mediated regulation of PPARG stability and expression. Mechanistically, NCK1 enhanced EIF3D phosphorylation by suppressing its O-GlcNAcylation.

As a molecular scaffold, NCK1 modulates protein translation through interactions with target proteins [24]. Previous studies have highlighted the immunomodulatory roles of NCK1 in Jurkat T cells [6, 8]. Recently, Ding et al. [10] reported that NCK1 is downregulated in the decidua of patients with recurrent pregnancy loss. *In vitro* studies revealed that NCK1 deficiency impaired trophoblast cell proliferation and migration, underscoring its critical role in pregnancy [10]. Given our observation that NCK1 was downregulated in PE, we hypothesized that NCK1 may be involved in the pathogenesis of PE. Functional analyses further demonstrated that NCK1 overexpression partially rescued hypoxia-induced defects in CTB syncytialization. Mechanistically, we found that overexpression of NCK1 enhanced both the stability and expression of PPARG. PPARG is a nuclear receptor that plays a key role in placental formation [25]. In the human placenta, knockdown of PPARG has been demonstrated to significantly impair STB formation by disrupting trophoblast fusion [15]. Consistently, our results demonstrated that upregulation of PPARG alleviated hypoxia-induced CTB fusion defects, whereas PPARG deficiency abolished the promoting effects of NCK1 on CTB syncytialization.

m6A is one of the most abundant RNA epigenetic modifications and is considered a promising new mechanism of post-transcriptional gene regulation [26]. Dysregulation of m6A has been implicated in various pathological conditions, including cancer [27], metabolic diseases [27], and cardiovascular disorders [28]. Recently, accumulating evidence has emphasized the critical role of m6A in uterine spiral artery remodeling [29, 30]. For example, m6A-deficient mice exhibit pronounced PE-like phenotypes [29]. Consistent with these findings, our data showed that hypoxia reduced m6A modification levels on PPARG mRNA. Guo et al. [14] reported that downregulation of ALKBH5, an m6A demethylase, significantly increased m6A levels on PPARG mRNA and enhanced its stability, thereby alleviating PE progression. Notably, our study provides the first evidence that NCK1 overexpression elevates m6A modification of PPARG mRNA, suggesting that its pro-syncytialization effects in CTB cells are mediated, at least in part, through PPARG regulation.

EIF3D, a non-canonical mRNA cap-binding protein, has been reported to be upregulated in placental tissues of patients with PE [12]. Elevated EIF3D expression promotes trophoblast cell proliferation and invasion, thereby exacerbating PE progression [12]. Mukhopadhyay et al. [13] demonstrated that sustained phosphorylation of EIF3D impairs 5′ cap recognition and subsequent translation of downstream transcripts. In the present study, we observed a significant increase in EIF3D phosphorylation during spontaneous syncytialization of CTB cells. Moreover, NCK1 overexpression partially rescued hypoxia-induced suppression of EIF3D phosphorylation, suggesting that NCK1 may exert protective effects against hypoxia-induced trophoblast fusion defects by modulating EIF3D phosphorylation status. Given that previous studies have shown that dephosphorylation of EIF3D significantly upregulates the expression of ALKBH5 [13], we hypothesized that NCK1 regulates PPARG stability by modulating EIF3D phosphorylation. Consistent with this hypothesis, knockdown of EIF3D markedly reduced m6A levels on PPARG mRNA, which may be attributed to impaired NCK1-mediated suppression of ALKBH5. Thus, these findings suggest that NCK1-mediated regulation of EIF3D phosphorylation may act as a molecular switch that regulates PPARG m6A modification and, consequently, mRNA stability.

Mechanistically, O-GlcNAcylation is a form of post-translational glycosylation in which O-linked N-acetylglucosamine (O-GlcNAc) is transferred from the donor substrate UDP-GlcNAc to Ser/Thr residues of target proteins by O-GlcNAc transferase (OGT) [31]. Notably, because both O-GlcNAcylation and phosphorylation occur on Ser/Thr residues, O-GlcNAcylation exhibits complex crosstalk with phosphorylation [32]. Previous studies have shown that NCK1 deficiency enhances PERK activation by increasing its phosphorylation [21]. Activated PERK can directly phosphorylate OGT, which in turn promotes glycosylation of its substrate Tomm70, ultimately enhancing mitochondrial cristae abundance [33]. In this study, we found that OGT-mediated suppression of EIF3D phosphorylation was reversed by NCK1 overexpression, suggesting that NCK1 enhances EIF3D phosphorylation by inhibiting its O-GlcNAcylation. Additionally, emerging evidence has linked O-GlcNAcylation to placental development under pathological conditions, including hypertension and stress-related disorders. For example, in PE placentas, impaired trophoblast fusion has been associated with O-GlcNAcylation of cystathionine γ-lyase [34]. Therefore, our findings demonstrate that NCK1 modulates the crosstalk between O-GlcNAcylation and phosphorylation status of EIF3D, thereby stabilizing PPARG and rescuing hypoxia-impaired syncytialization in CTB cells.

One limitation of the present study is that the proteomic comparison was performed using placentas from patients with early-onset PE and normal term pregnancies. Since placental protein expression changes dynamically across gestation, gestational age may represent a potential confounding factor when interpreting the DEPs. Thus, although our proteomic data provide useful insights into molecular alterations associated with PE, some changes may reflect, at least in part, gestational age-related differences rather than disease-specific effects alone.

**Conclusions**

In summary, NCK1 expression was markedly downregulated in placentas from patients with PE and in hypoxia-exposed CTB cells. Overexpression of NCK1 alleviated hypoxia-induced defects in CTB cell fusion. Mechanistically, NCK1 enhanced m6A modification of PPARG mRNA, thereby increasing its stability. Furthermore, NCK1 promoted EIF3D phosphorylation by inhibiting its O-GlcNAcylation, leading to upregulation of PPARG protein expression. Collectively, our work uncovers a previously unrecognized role of NCK1 in the pathophysiology of PE and identifies the NCK1/EIF3D/PPARG axis as a molecular pathway potentially involved in trophoblast fusion dysfunction in PE.

**List of abbreviations**

ALKBH5, alpha-ketoglutarate-dependent homolog 5

CTB, cytotrophoblast

EIF3D, Eukaryotic Translation Initiation Factor 3 Subunit

FBS, fetal bovine serum

NCK1, NCK Adaptor Protein 1

O-GlcNAc, O-GlcNAcylation

PBMCs, peripheral blood mononuclear cells

PE, preeclampsia

PPARG, Peroxisome Proliferator-Activated Receptor Gamma

STB, syncytiotrophoblast

**Declarations**

**Ethics approval and consent to participate**

This study was approved by the Ethics Committee of Shengjing Hospital of China Medical University. Informed consent was obtained from all individual participants included in the study.

**Consent for publication**

Not applicable.

**Availability of data and materials**

The datasets generated and analyzed during the current study are available from the ProteomeXchange Consortium (https://proteomecentral.proteomexchange.org) via the iProX partner repository under the dataset identifier PXD075843.

**Competing interests**

The authors declare that they have no competing interests.

**Funding**

This work was supported by the Liaoning Provincial Natural Science Foundation of China (grant number 2023-MS-155).

**Authors’ contributions**

YS and XG designed the study, developed the experimental protocol, and acquired the data. YS wrote the initial manuscript. XG, JW, and YH revised the article critically for important intellectual content. All authors reviewed and approved the final manuscript.

**Acknowledgements**

Not applicable.

**References**

1.	Burton GJ, Redman CW, Roberts JM and Moffett A (2019) Pre-eclampsia: pathophysiology and clinical implications. Bmj 366:l2381. doi: 10.1136/bmj.l2381

2.	Yung HW, Colleoni F, Dommett E, Cindrova-Davies T, Kingdom J, Murray AJ, et al. (2019) Noncanonical mitochondrial unfolded protein response impairs placental oxidative phosphorylation in early-onset preeclampsia. Proc Natl Acad Sci U S A 116:18109-18118. doi: 10.1073/pnas.1907548116

3.	Carrasco-Wong I, Aguilera-Olguín M, Escalona-Rivano R, Chiarello DI, Barragán-Zúñiga LJ, Sosa-Macías M, et al. (2021) Syncytiotrophoblast stress in early onset preeclampsia: The issues perpetuating the syndrome. Placenta 113:57-66. doi: 10.1016/j.placenta.2021.05.002

4.	Mukherjee I, Dhar R, Singh S, Sharma JB, Nag TC, Mridha AR, et al. (2021) Oxidative stress-induced impairment of trophoblast function causes preeclampsia through the unfolded protein response pathway. Sci Rep 11:18415. doi: 10.1038/s41598-021-97799-y

5.	Colson A, Depoix CL, Baldin P, Hubinont C, Sonveaux P and Debiève F (2020) Hypoxia-inducible factor 2 alpha impairs human cytotrophoblast syncytialization: New insights into placental dysfunction and fetal growth restriction. Faseb j 34:15222-15235. doi: 10.1096/fj.202001681R

6.	Paensuwan P, Hartl FA, Yousefi OS, Ngoenkam J, Wipa P, Beck-Garcia E, et al. (2016) Correction: Nck Binds to the T Cell Antigen Receptor Using Its SH3.1 and SH2 Domains in a Cooperative Manner, Promoting TCR Functioning. J Immunol 196:4833. doi: 10.4049/jimmunol.1600551

7.	Ngoenkam J, Paensuwan P, Preechanukul K, Khamsri B, Yiemwattana I, Beck-García E, et al. (2014) Non-overlapping functions of Nck1 and Nck2 adaptor proteins in T cell activation. Cell Commun Signal 12:21. doi: 10.1186/1478-811x-12-21

8.	Roy E, Togbe D, Holdorf A, Trubetskoy D, Nabti S, Küblbeck G, et al. (2010) Fine tuning of the threshold of T cell selection by the Nck adapters. J Immunol 185:7518-26. doi: 10.4049/jimmunol.1000008

9.	He P, Sheng J, Qi J, Bai X, Li J, Wang F, et al. (2022) STAT3-induced NCK1 elevation promotes migration of triple-negative breast cancer cells via regulating ERK1/2 signaling. Mol Biol Rep 49:267-278. doi: 10.1007/s11033-021-06868-y

10.	Ding C, Zhang D, Bao S, Zhao X, Yu Y and Zhou Q (2023) Reduced NCK1 participates in unexplained recurrent miscarriage by regulating trophoblast functions and macrophage proliferation at maternal-fetal interface. Genet Mol Biol 46:e20220297. doi: 10.1590/1678-4685-gmb-2022-0297

11.	Lee AS, Kranzusch PJ, Doudna JA and Cate JH (2016) eIF3d is an mRNA cap-binding protein that is required for specialized translation initiation. Nature 536:96-9. doi: 10.1038/nature18954

12.	Li X, Wang Z, Liu G and Guo J (2021) EIF3D promotes the progression of preeclampsia by inhibiting of MAPK/ERK1/2 pathway. Reprod Toxicol 105:166-174. doi: 10.1016/j.reprotox.2021.09.006

13.	Mukhopadhyay S, Amodeo ME and Lee ASY (2023) eIF3d controls the persistent integrated stress response. Mol Cell 83:3303-3313.e6. doi: 10.1016/j.molcel.2023.08.008

14.	Guo Y, Song W and Yang Y (2022) Inhibition of ALKBH5-mediated m(6) A modification of PPARG mRNA alleviates H/R-induced oxidative stress and apoptosis in placenta trophoblast. Environ Toxicol 37:910-924. doi: 10.1002/tox.23454

15.	Shoaito H, Chauveau S, Gosseaume C, Bourguet W, Vigouroux C, Vatier C, et al. (2020) Peroxisome proliferator-activated receptor gamma-ligand-binding domain mutations associated with familial partial lipodystrophy type 3 disrupt human trophoblast fusion and fibroblast migration. J Cell Mol Med 24:7660-7669. doi: 10.1111/jcmm.15401

16.	Jaju Bhattad G, Jeyarajah MJ, McGill MG, Dumeaux V, Okae H, Arima T, et al. (2020) Histone deacetylase 1 and 2 drive differentiation and fusion of progenitor cells in human placental trophoblasts. Cell Death Dis 11:311. doi: 10.1038/s41419-020-2500-6

17.	Chen L, Zhou Q, Zhang P, Tan W, Li Y, Xu Z, et al. (2024) Direct stimulation of de novo nucleotide synthesis by O-GlcNAcylation. Nat Chem Biol 20:19-29. doi: 10.1038/s41589-023-01354-x

18.	Sun D, Zhu H, Ai L, Wu H, Wu Y and Jin J (2021) Mitochondrial fusion protein 2 regulates endoplasmic reticulum stress in preeclampsia. J Zhejiang Univ Sci B 22:165-170. doi: 10.1631/jzus.B2000557

19.	Lorenzon-Ojea AR, Yung HW, Burton GJ and Bevilacqua E (2020) The potential contribution of stromal cell-derived factor 2 (SDF2) in endoplasmic reticulum stress response in severe preeclampsia and labor-onset. Biochim Biophys Acta Mol Basis Dis 1866:165386. doi: 10.1016/j.bbadis.2019.01.012

20.	Bolnick AD, Bolnick JM, Kohan-Ghadr HR, Kilburn BA, Pasalodos OJ, Singhal PK, et al. (2017) Enhancement of trophoblast differentiation and survival by low molecular weight heparin requires heparin-binding EGF-like growth factor. Hum Reprod 32:1218-1229. doi: 10.1093/humrep/dex069

21.	Yamani L, Latreille M and Larose L (2014) Interaction of Nck1 and PERK phosphorylated at Y⁵⁶¹ negatively modulates PERK activity and PERK regulation of pancreatic β-cell proinsulin content. Mol Biol Cell 25:702-11. doi: 10.1091/mbc.E13-09-0511

22.	Steentoft C, Vakhrushev SY, Joshi HJ, Kong Y, Vester-Christensen MB, Schjoldager KT, et al. (2013) Precision mapping of the human O-GalNAc glycoproteome through SimpleCell technology. Embo j 32:1478-88. doi: 10.1038/emboj.2013.79

23.	Xue Y, Yang N, Gu X, Wang Y, Zhang H and Jia K (2023) Risk Prediction Model of Early-Onset Preeclampsia Based on Risk Factors and Routine Laboratory Indicators. Life (Basel) 13. doi: 10.3390/life13081648

24.	Kebache S, Zuo D, Chevet E and Larose L (2002) Modulation of protein translation by Nck-1. Proc Natl Acad Sci U S A 99:5406-11. doi: 10.1073/pnas.082483399

25.	Barak Y, Nelson MC, Ong ES, Jones YZ, Ruiz-Lozano P, Chien KR, et al. (1999) PPAR gamma is required for placental, cardiac, and adipose tissue development. Mol Cell 4:585-95. doi: 10.1016/s1097-2765(00)80209-9

26.	Zhang C, Fu J and Zhou Y (2019) A Review in Research Progress Concerning m6A Methylation and Immunoregulation. Front Immunol 10:922. doi: 10.3389/fimmu.2019.00922

27.	An Y and Duan H (2022) The role of m6A RNA methylation in cancer metabolism. Mol Cancer 21:14. doi: 10.1186/s12943-022-01500-4

28.	Kumari R, Ranjan P, Suleiman ZG, Goswami SK, Li J, Prasad R, et al. (2022) mRNA modifications in cardiovascular biology and disease: with a focus on m6A modification. Cardiovasc Res 118:1680-1692. doi: 10.1093/cvr/cvab160

29.	Liu S, Chen W, Chen J, Liu T, Deng M, Xia L, et al. (2025) m(6)A deficiency impairs uterine spiral artery remodeling to induce preeclampsia-like symptoms via FGF2. Sci China Life Sci. doi: 10.1007/s11427-024-2846-4

30.	Wang Y, Zhang G, Gao Y, Zhang X and Qi H (2024) METTL3 promotes trophoblast ferroptosis in preeclampsia by stabilizing the ACSL4 m(6)A modification. Exp Cell Res 437:113990. doi: 10.1016/j.yexcr.2024.113990

31.	Holt GD, Snow CM, Senior A, Haltiwanger RS, Gerace L and Hart GW (1987) Nuclear pore complex glycoproteins contain cytoplasmically disposed O-linked N-acetylglucosamine. J Cell Biol 104:1157-64. doi: 10.1083/jcb.104.5.1157

32.	Chang YH, Weng CL and Lin KI (2020) O-GlcNAcylation and its role in the immune system. J Biomed Sci 27:57. doi: 10.1186/s12929-020-00648-9

33.	Latorre-Muro P, O'Malley KE, Bennett CF, Perry EA, Balsa E, Tavares CDJ, et al. (2021) A cold-stress-inducible PERK/OGT axis controls TOM70-assisted mitochondrial protein import and cristae formation. Cell Metab 33:598-614.e7. doi: 10.1016/j.cmet.2021.01.013

34.	Liu J, Shao X, Qin W, Zhang Y, Dang F, Yang Q, et al. (2021) Quantitative chemoproteomics reveals O-GlcNAcylation of cystathionine γ-lyase (CSE) represses trophoblast syncytialization. Cell Chem Biol 28:788-801.e5. doi: 10.1016/j.chembiol.2021.01.024

**Figure legends**

**Figure 1** The protein expression profile in placental tissues from patients with early-onset PE. **(A)** PCA plot of samples. **(B)** Volcano plot showing differentially expressed proteins (DEPs) in placental tissue from PE patients versus normal term placentas. **(C)** Gene Ontology biological process (GO-BP) and KEGG pathway enrichment analysis of downregulated DEPs. **(D)** Venn diagram of shared candidates. Proteins were selected from the GeneCards database (https://www.genecards.org/) using the keyword “trophoblast fusion” with a selection threshold of relevance score > median. The heatmap shows the downregulated proteins in placental tissues from patients with PE. **(E)** Expression of NCK1 was assessed by immunoblotting (n = 6 biological replicates).

**Figure** **2** The expression of NCK1 during syncytialization of primary CTB cells. **(A)** The expression pattern of NCK1 in normal term placenta was detected by immunohistochemistry. CK7-positive cells indicate the CTB cells. Arrowheads indicate syncytial knots in the STB. **(B)** Representative immunofluorescence images and corresponding bright-field images showing the E-cadherin expression in primary CTB cells cultured for 0 and 5 days. Dotted areas indicate syncytia formation in CTB cells, and arrows mark single cells (S). **(C)** ELISA detection of β-hCG in cell supernatant (n = 3 biological replicates, each with three technical replicates). **(D-F)** The mRNA levels of CGB, ERVW1 and ERVFRD-1 were measured by qPCR (n = 3 biological replicates, each with three technical replicates). **(G)** Expression of NCK1 was assessed by immunoblotting (n = 3 biological replicates).

**Figure** **3** Effects of NCK1 on primary CTB cell fusion under hypoxia. Primary CTB cells were cultured for 5 days under normoxic or hypoxic conditions. **(A)** E-cadherin expression is shown in representative immunofluorescence images and corresponding bright-field images. Dotted areas indicate syncytia formation in CTB cells, and arrows mark single cells (S). **(B)** The expression of β-hCG in primary CTB cells was measured by immunoblotting analysis (n = 3 biological replicates). **(C-E)** The mRNA levels of CGB, ERVW1 and ERVFRD-1 in primary CTB cells were measured by qPCR (n = 3 biological replicates, each with three technical replicates). **(F)** NCK1 expression in primary CTB cells was measured by immunoblotting analysis (n = 3 biological replicates). Primary CTB cells were infected with the indicated lentiviral particles. Forty-eight hours later, the cells were cultured under hypoxic conditions for 72 h. **(G)** NCK1 expression was assessed by immunoblotting analysis (n = 3 biological replicates). **(H)** E-cadherin expression was shown in representative immunofluorescence images and corresponding bright-field images. Dotted areas indicate syncytia formation in CTB cells, and arrows mark single cells (S). **(I)** β-hCG expression was measured by immunoblotting analysis (n = 3 biological replicates). **(J-L)** The mRNA levels of CGB, ERVW1 and ERVFRD-1 were measured by qPCR (n = 3 biological replicates, each with three technical replicates).

**Figure** **4** Effects of NCK1 on PPARG expression during primary CTB cell fusion. **(A)** Expression of PPARG during syncytialization of primary CTB cells (n = 3 biological replicates). Primary CTB cells were infected with the indicated lentiviral particles. Forty-eight hours post-infection, cells were cultured for 72 hunder normoxic or hypoxic conditions. **(B, C)** Expression of PPARG was assessed by qPCR (n = 3 biological replicates, each with three technical replicates) and immunoblotting analysis (n = 3 biological replicates). **(D)** To evaluate mRNA stability, primary CTB cells were infected with lentivirus for 48 hours, cultured under normoxia or hypoxia for an additional 72 h, and then treated with actinomycin D (5 μg/ml) to inhibit transcription. PPARG mRNA levels were measured by qPCR at 0, 2, 4, and 6 hours after actinomycin D treatment (n = 3 biological replicates, each with three technical replicates). **(E)** Changes in m6A-modified PPARG mRNA levels upon NCK1 overexpression (n = 3 biological replicates, each with three technical replicates).

**Figure** **5** PPARG-mediated effects of NCK1 on CTB cell fusion. Primary CTB cells were infected with the indicated lentiviral particles. Forty-eight hours post-infection, cells were cultured for 72 hours under normoxic or hypoxic conditions. **(A)** PPARG mRNA levels were assessed by qPCR (n = 3 biological replicates, each with three technical replicates). **(B)** E-cadherin expression is shown in representative immunofluorescence images and corresponding bright-field images. Dotted areas indicate syncytia formation in CTB cells, and arrows mark single cells (S). **(C)** ERVFRD-1 mRNA levels were measured by qPCR (n = 3 biological replicates, each with three technical replicates). **(D)** β-hCG expression was measured by immunoblotting analysis (n = 3 biological replicates). For hypoxia-specific experiments, primary CTB cells were infected with the indicated lentiviral particles, cultured for 48 hours, and then maintained under hypoxic conditions for 72 hours. **(E, F)** PPARG and ERVFRD-1 mRNA levels were measured by qPCR (n = 3 biological replicates, each with three technical replicates). **(G)** β-hCG expression was assessed by immunoblotting analysis (n = 3 biological replicates). **(H)** E-cadherin expression was shown in representative immunofluorescence images and corresponding bright-field images. Dotted areas indicate syncytia formation in CTB cells, and arrows mark single cells (S).

**Figure** **6** EIF3D mediates the regulation of PPARG expression by NCK1. **(A)** Phosphorylation of EIF3D during syncytialization of primary CTB cells was assessed by Co-IP assay (n = 3 biological replicates). Primary CTB cells were infected with the indicated lentiviral particles. Forty-eight hours post-infection, cells were cultured for 72 hours under normoxic or hypoxic conditions. **(B)** Phosphorylation of EIF3D was detected by Co-IP assay. **(C)** EIF3D expression in primary CTB cells infected with the indicated lentiviral particles was assessed by immunoblotting 48 hours post-infection (n = 3 biological replicates). For hypoxia-specific experiments, cells were cultured under hypoxic condition for 72 hours. **(D)** PPARG mRNA levels in the indicated CTB cells were measured by qPCR at 0, 2, 4 and 6 hours after actinomycin D treatment (n = 3 biological replicates, each with three technical replicates). **(E)** Changes in m6A-modified PPARG mRNA levels were detected by qPCR (n = 3 biological replicates, each with three technical replicates). **(F)** ALKBH5 expression was assessed by immunoblotting analysis (n = 3 biological replicates).

**Figure** **7** NCK1 modulates the crosstalk between O-GlcNAcylation and phosphorylation of EIF3D. **(A)** EIF3D O-GlcNAcylation during syncytialization of primary CTB cells was detected by Co-IP assay. Primary CTB cells were infected with the indicated lentiviral particles. Forty-eight hours post-infection, cells were cultured for 72 hours under hypoxic conditions unless otherwise specified. **(B)** EIF3D O-GlcNAcylation in the indicated CTB cells was analyzed using a chemoenzymatic labeling method under normoxic or hypoxic conditions. **(C)** The levels of EIF3D and EIF3D O-GlcNAcylation were assessed by Co-IP assay. **(D, E)** HEK293T cells were transfected with the indicated plasmids and analyzed 48 hours post-transfection. The levels of Flag-tagged EIF3D, EIF3D O-GlcNAcylation, and EIF3D phosphorylation were measured by Co-IP assay. O-GlcNAc, O-GlcNAcylation.
