# Supplementary material for: Regulation of trophoblast fusion dysfunction in preeclampsia by the NCK1/EIF3D/PPARG axis
Source: Biol Res. 2026 Jun 10;59:46. doi: 10.1186/s40659-026-00704-y (PMC13479735; doi:10.1186/s40659-026-00704-y)
Supplement: Supplementary file 2 [file 40659_2026_704_MOESM2_ESM.docx]

BRES-D-25-00713R1
Regulation of Trophoblast Fusion Dysfunction in Preeclampsia by the NCK1/EIF3D/PPARG Axis
Ying Shen; Xiaolin Gao; Jiashi Wang; Yingying Hao
Biological Research

Dear Editor,
We greatly appreciate the opportunity to revise our work for consideration in *Biological Researc*h.

We have carefully addressed all editorial comments and revised the manuscript accordingly. Detailed point-by-point responses to the reviewers’ comments are provided below.

We hope that the revised manuscript meets the standards of *Biological Research* and is now suitable for publication.

Thank you again for your time and consideration.

Sincerely,

Dr. Yingying Hao

E-mail: haoyy_doc@163.com

**Point-by-point response to the reviewer comments**

Dear Dr. Hao,

Your manuscript 'Regulation of Trophoblast Fusion Dysfunction in Preeclampsia by the NCK1/EIF3D/PPARG Axis' (BRES-D-25-00713R1) has been assessed by our reviewers.

After evaluating the revised manuscript and the reviewers’ comments, I am pleased to inform you that the manuscript is potentially suitable for acceptance pending minor revision. The revised version has addressed the main concerns raised during peer review, including the rationale for candidate selection, clarification of the PPARG expression results under hypoxia, deposition of the proteomic dataset, improved description of qPCR replication, and revision of the figures.

Before we can proceed further, please address the following editorial points:

First, please add a brief limitation to the Discussion acknowledging that the proteomic comparison was performed using placentas from early-onset preeclampsia and normal term placentas. Because gestational age may influence placental protein expression, this should be explicitly recognized as a potential confounding factor in the interpretation of the proteomic findings.

Second, please moderate the therapeutic wording throughout the manuscript. Statements such as “potential therapeutic target for PE” should be revised to better reflect the experimental scope of the study. A more appropriate formulation would be that the NCK1/EIF3D/PPARG axis represents a molecular pathway potentially relevant to trophoblast fusion dysfunction in preeclampsia. The current data support mechanistic relevance in placental tissue and trophoblast models, but not yet therapeutic efficacy.

Third, please ensure that the proteomic data availability statement is complete and that the ProteomeXchange/iProX accession number and reviewer access information are correct and functional. The final manuscript should clearly indicate the dataset identifier and repository.
Finally, please carefully review the manuscript for minor language and typographical errors before submitting the final version.

**Response:**

Thank you very much for your comments and suggestions. We have carefully revised the manuscript accordingly, and the relevant changes are highlighted in red in the revised version. Our point-by-point responses are provided below.

**Comment 1:**

We agree that gestational age may influence placental protein expression and should be acknowledged as a potential confounding factor. We have added a statement in the Discussion to clarify this limitation and to caution the interpretation of the proteomic findings. Please see the revised Discussion section, Page 24, Lines 492–498.

**Comment 2:**

Thank you for this helpful comment. We have revised the relevant wording throughout the manuscript to avoid overstatement. In particular, expressions such as “potential therapeutic target for PE” have been modified to more accurately reflect the experimental scope of this study. The revised text now describes the NCK1/EIF3D/PPARG axis as a molecular pathway potentially relevant to trophoblast fusion dysfunction in PE, rather than implying therapeutic efficacy. These changes have been made in the Abstract (page 3), Background (page 6), and Conclusion (page 24) sections, as highlighted in red in the revised manuscript.

**Comment 3:**

We have updated the proteomic data availability statement. The dataset identifier and repository are now clearly indicated in the revised manuscript.

Additionally, we have carefully reviewed the entire manuscript and corrected minor language and typographical errors throughout the revised version.
